# In Vitro Thermodynamic Dissection of Human Copper Transfer from Chaperone to Target Protein

**DOI:** 10.1371/journal.pone.0036102

**Published:** 2012-05-04

**Authors:** Moritz S. Niemiec, Christoph F. Weise, Pernilla Wittung-Stafshede

**Affiliations:** Chemistry Department, Chemical Biological Center, Umeå University, Umeå, Sweden; Russian Academy of Sciences, Institute for Biological Instrumentation, Russian Federation

## Abstract

Transient protein-protein and protein-ligand interactions are fundamental components of biological activity. To understand biological activity, not only the structures of the involved proteins are important but also the energetics of the individual steps of a reaction. Here we use in vitro biophysical methods to deduce thermodynamic parameters of copper (Cu) transfer from the human copper chaperone Atox1 to the fourth metal-binding domain of the Wilson disease protein (WD4). Atox1 and WD4 have the same fold (ferredoxin-like fold) and Cu-binding site (two surface exposed cysteine residues) and thus it is not clear what drives metal transfer from one protein to the other. Cu transfer is a two-step reaction involving a metal-dependent ternary complex in which the metal is coordinated by cysteines from both proteins (i.e., Atox1-Cu-WD4). We employ size exclusion chromatography to estimate individual equilibrium constants for the two steps. This information together with calorimetric titration data are used to reveal enthalpic and entropic contributions of each step in the transfer process. Upon combining the equilibrium constants for both steps, a metal exchange factor (from Atox1 to WD4) of 10 is calculated, governed by a negative net enthalpy change of ∼10 kJ/mol. Thus, small variations in interaction energies, not always obvious upon comparing protein structures alone, may fuel vectorial metal transfer.

## Introduction

Protein-protein interactions and protein-ligand interactions are responsible for most biological functions. The development of protein structure determination by NMR and crystallography has improved our understanding of protein function dramatically. However, to pinpoint why and how biological reactions occur it is necessary to identify the thermodynamic and kinetic driving forces. Formation of protein-protein and protein-ligand complexes are often favored thermodynamically (that is, the free energy of complex formation is negative) due to an increase in negative enthalpy or positive entropy, or a combination of both. For example, the assembly of monomers into the functional heptameric co-chaperonin protein 10 is entropically driven [1] whereas many protein-DNA complexes are associated with favorable enthalpy changes [2]. In many signaling and transport pathways in living systems, protein-protein and protein-ligand complexes are formed transiently followed by dissociation resulting in a vectorial transfer of a ligand or a signal. In such cases it is not always easy to deduce the energetic components involved in every step of the path as pure intermediates are hard to isolate and reactants and products often have similar properties. In the respiration chain, electrons flow between metal centers in membrane proteins with varying redox potentials resulting in an electrostatic potential across the membrane. In human copper transport, copper is shuttled from one protein to another to allow entry into the Golgi network where loading of copper-dependent enzymes in the secretory pathway occur [3], [4], [5]. Despite structural work on the proteins involved in this chain, it is not clear what the driving force for vectorial copper transfer is as the involved proteins have similar folds and Cu-binding sites.

**Figure 1:**
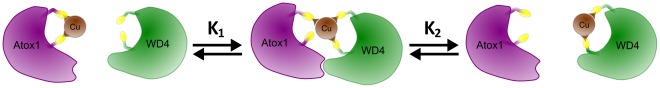
Illustration of Scheme 1. Upon mixing Cu-loaded Atox1 (purple) and apo-WD (green), the proteins interact and form a hetero-complex, Atox1-Cu-WD4 (shown with Cu coordinating one Cys in Atox1 and both Cys in WD4; however, there are other possible Cu coordinations in the hetero-complex [17]), and also products, apo-Atox1 and Cu-WD4, according to the equilibrium constants K_1_ and K_2_.

Cu is found in the active sites of proteins that participate in cellular reactions such as respiration, antioxidant defense, neurotransmitter biosynthesis, connective-tissue biosynthesis and pigment formation [6], [7], [8]. The thermodynamic and kinetic feasibility of Cu to oxidize/reduce (switching between Cu^1+^ and Cu^2+^) allows copper-containing proteins to play important roles as electron carriers and redox catalysts in living systems. To avoid toxicity and to overcome solubility problems of Cu^1+^, the intracellular concentration of Cu is regulated *via* dedicated proteins that facilitate its uptake, efflux as well as distribution to target Cu-dependent proteins and enzymes [3], [4], [5]. In humans, the 68-residue Cu chaperone Atox1 picks up Cu that has entered the cell via CTR1 and delivers the metal to cytoplasmic metal-binding domains in ATP7A and ATP7B (also called Menkes and Wilson disease proteins), two homologous multi-domain P_1B_-type ATPases located in the trans-Golgi network. Many human copper-dependent enzymes (*e.g.,* blood clotting factors, tyrosinase, lysyl oxidase and ceruloplasmin) acquire Cu in the Golgi before reaching their final destination [3], [4], [5]. In both ATP7A and ATP7B, there are six metal-binding domains in the N-terminal cytoplasmic part separated by peptide linkers [9]. Structural work has demonstrated that Cu chaperones and target domains from many different organisms possess the same ferredoxin-like fold and Cu-binding motif [10]. The six domains, as well as Atox1, bind Cu via two conserved Cys residues in a surface-exposed MxCxxC copper-binding motif [6], [10]. Earlier *in vitro* [11], [12], [13], [14], [15], [16] and *in silico* [17] work has shown that Cu transfer from Atox1 to metal-binding domains (WD) of the Wilson disease protein and metal-binding domains (MK) of the Menkes protein proceeds via a copper-bridged hetero-dimer complex where the metal is shared between the two metal-binding sites (**Figure 1**).

### Scheme 1

During this process, Cu is thought to undergo a series of rapid associative exchange reactions involving two- or three-coordinated Cu-sulfur intermediates that ultimately allows movement of the Cu ion from one protein site to another [14]. NMR experiments have shown that the possibility to detect the hetero-complex in mixing experiments depends on which target WD or MK domain is studied [11], [12], [18], [19]. When such hetero-complexes are detected by NMR, most often via slowed tumbling times, they are found to be in fast exchange with the free proteins [11]. Based on affinity and NMR studies, Cu binding to a Wilson disease domain is favored over binding to Atox1 by a factor of 3–5 [18], [20], [21]. In agreement with NMR data [18], we showed earlier that upon mixing of Cu-Atox1 and the fourth metal-binding domain of Wilson disease protein (WD4), a stable ternary complex could be inferred from the near-UV CD spectrum [22]. However, the ternary complex was in equilibrium with both substrate and products of the overall reaction *scheme 1*. Here we dissect the reaction in *scheme 1* to reveal energetic components of each of the individual steps. It emerges that vectorial Cu transfer (towards the Golgi) is enthalpically driven.

## Results

### Detecting the Hetero-complex

**Figure 2:**
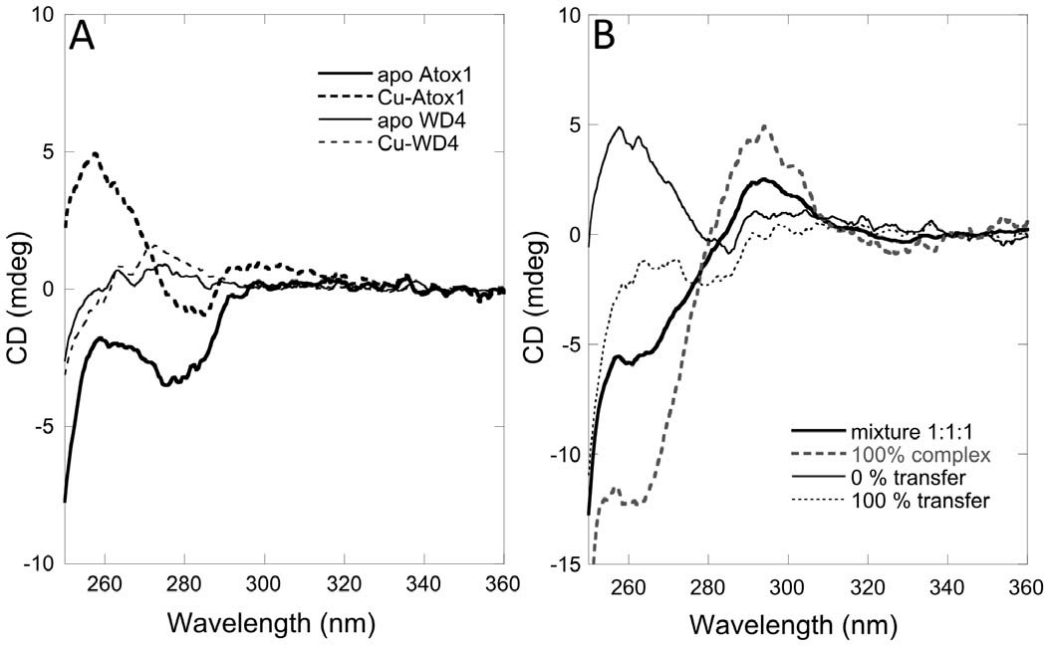
CD reveals ternary complex. A. Near-UV CD of apo and holo-forms of Atox1 and WD4. B. Near-UV CD of a 1-to-1 mixture of Cu-Atox1 and apo-WD. For comparison the theoretical CD signal derived for no reaction (i.e., sum of Cu-Atox1 and apo-WD4 signals) and for 100% reaction (i.e., signals for apo-Atox1 and holo-WD4) are also shown. The dotted line is the calculated CD signal for 100% pure heterocomplex at the same concentration (see text).

The near-UV CD region is strongly influenced by ligand-to-metal-charge-transfer from Cys-Cu bonds and has been used as a method to study the metal environment in metallothioneins which also binds Cu via Cys thiols [23], [24]. In accord, for both Atox1 and WD4, addition of Cu to the apo-protein results in distinct near-UV CD changes (**Figure 2A** **)**. Combination of spectra for apo- and holo-forms was used to generate theoretical signals for 0 and 100% transfer of Cu from Atox1 to WD4. However, when Cu-Atox1 is mixed with apo-WD4 the resulting near-UV spectrum does not match that for Cu transfer; instead, the new signal is distinct, with a positive peak at 295 nm and a more negative signal at 265 nm (**Figure 2B**), as previously reported [22]. This indicates the formation of a protein-protein complex that contains a unique Cu site (*i.e.,* 3 or 4 Cys ligands instead of 2). Formation of a ternary complex was also reported from NMR experiments [15], [16]. However, the CD data does not resolve how much of each of the species in *scheme 1* is present in the solution. It is a combination of reactants, intermediate and products in equilibrium with each other that depends on K_1_ and K_2_. To resolve the concentrations of the various species that are present, and thereby deduce the equilibrium constants K_1_ and K_2_, we turned to size exclusion chromatography (SEC).

### Separating Species with SEC

**Figure 3:**
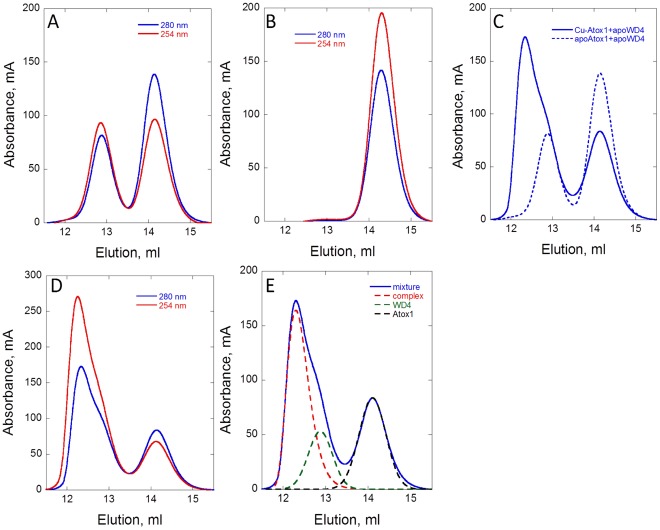
SEC probed at 2 wavelengths is used to separate equilibrium species in Scheme 1. **A.** SEC analysis of a mixture of the two apo proteins (300 µM each) at 280 and 254 nm. **B.** SEC analysis of holo Atox1 (300 µM) at 280 and 254 nm. **C.** SEC analysis at 280 nm of a mixture of 300 µM Cu-Atox1 and 300 µM apo-WD4. For comparison, the 280 nm elution trace for the mixture of the two apo-proteins is shown. **D.** SEC analysis at 280 nm and 254 nm of a mixture of 300 µM Cu-Atox1 and 300 µM apo-WD4. **E.** De-convolution of the underlying peaks in the elution trace of the Cu-Atox1+apo-WD4 mixture.

SEC of a mixture of the apo proteins is shown in **Figure 3A** and of the two holo proteins in **Figure 3BC**. Elution of the proteins was followed by absorption at 280 nm (reporting on amount of protein) and at 254 nm (reporting on Cu-loading). It is clear from the data in **Figures 3ABC** that the holo forms have higher absorption at 254 nm than the apo forms due to Cu-Cys transitions, although the 280 nm signal does not change. Absorption spectra confirm this (**Figure S1**) and from the data we can calculate extinction coefficients at 254 nm for apo respective holo forms. Despite the similar size and structure, the two proteins elute at different elution volumes. Specifically, WD4 elutes at an apparent higher molecular weight than expected for the size of the protein. We found that the elution volume of WD4 was salt dependent (not shown). Abnormal elution of copper chaperones has been noted before for CopZ [25]. To assure that WD4 was monomeric at low and high salt concentrations, we performed NMR diffusion experiments (**Table S1**). The results demonstrate that the apo- and holo-form of WD4 is monomeric at both salt conditions. The relative standard deviation between the four measurements reported in **Table S1** is similar to the experimental uncertainties, and suggests that the diameter of W4 does not change between apo and holo states, or upon addition of salt. The diffusion coefficient of an ideal sphere diffusing under Stokes-Einstein conditions is expected to decrease by ∼26% upon dimerization. Using the Cd-(Atox1)_2_ structure (1fe0.pdb) we computed a diffusion coefficient of 5.7*10^-11^ m^2^/s for an Atox1 dimer, this value is 23% smaller than the value found in the experiments and well below the uncertainty in D. Thus, we conclude that WD4 elutes as a monomer but that other factors such as surface charge alters its elution volume.

SEC of a mixture of Cu-Atox1 and apo-WD4 results in a different elution profile with two overlapping bands near the position of the individual WD4 band in addition to the Atox1 band (**Figure 3D**). De-convolution of the absorption profile at 280 nm reveals the presence of two Gaussian-shaped bands (for Atox1 and WD4) as well as a new feature that is asymmetrical that is proposed to correspond to the hetero-complex (**Figure 3D**). Asymmetrical band broadening has been noted in SEC examinations of dimer-monomer equilibria [26]. Analysis of the Atox1 band reveals that there is a lower Atox1 concentration in this peak than expected based on elution of the protein alone (or when mixing the two apo forms). This suggests that the missing Atox1 is engaged in a hetero-complex that elutes at a different volume than the monomer. To confirm that the new absorption feature is due to a hetero-complex, the peak content was analyzed by mass spectrometry. As expected, we find both Atox1 and WD4 in the early elution samples (**Figure S2A**). Moreover, native gel analysis of this mixture directly visualizes the existence of a stable protein-protein complex in the presence of Cu (**Figure S2B**). In agreement with thermodynamic expectations, mixing of higher concentrations of proteins results in a larger fraction of the hetero-complex peak (**Figure S3**).

### Equilibrium Constants from SEC Data

Assuming that *scheme 1* is correct and that equilibrium is established on the SEC column, we can use the information in the Atox1 peak to reveal the concentrations of all five species in *scheme 1*. First, the Atox1 peak itself determines the concentration of apo and Cu forms of Atox1 in the mixture (from the 280/254 absorption ratio). The remaining fraction of Atox1 must then be found in the Atox1-Cu-WD4 heterocomplex. This value dictates how much Cu is left (i.e., not in Cu-Atox1 or in the heterocomplex) and that defines the concentration of Cu-WD4. The remaining amount of WD4, not found in the hetero-complex or in Cu-WD4, is used to derive the concentration of apo-WD4. With this, the concentration of each species is determined and these can be used to calculate the equilibrium constants K_1_ and K_2_. This analysis was performed at three different starting concentrations of 1-to-1 Cu-Atox1:apo-WD4 mixtures (**Figure S3**) and the results are reported in **Table 1**. Importantly, when we analyzed a mixture of Cu-WD4 and apo-Atox1, the same SEC profile was found as when starting with Cu-Atox1 and apo-WD4 (**Figure S3**). This assures that equilibrium is established in the loaded solution during the filtration experiments. Moreover, analysis of mixing of Cu-Atox1 and apo-WD4 at one specific concentration but at varying salt (50, 100, 150 and 200 mM) no significant differences or trends are noted (data not shown). This suggests that in this range of salt concentrations, that include physiological conditions, the reaction occurs and, if charge-charge interactions are present in the complex they are not abolished by 200 mM salt. This was supported by comparing near-UV CD spectra at high and low salt (Figure S4). The average K_1_ and K_2_ values are 0.4*10^6^ M^–1^ (1/K_1_ = 2 µM) and 2.6*10^–5^ M (26 µM) which results in a metal exchange factor, K_1_*K_2_ of 11. This means that step 1 corresponds to a ΔG change of –30 kJ/mol and step 2 of+25 kJ/mol; resulting in an overall driving force for vectorial transfer towards WD4 of –5 kJ/mol.

**Table 1:** SEC analysis of concentrations of species.

Starting 1∶1:1 concentration	Cu-Atox1 (µM)	ApoWD4(µM)	Complex (µM)	ApoAtox1 (µM)	Cu-WD4 (µM)	% complex of total Cu	K_1_(M^–1^)	K_2_(M)	K_1_*K_2_
75 µM	1.59	4.19	1.88	6.26	6.26	9.3	0.28*106	20.9*10–6	6
75 µM (opposite)	1.45	4.04	2.32	5.96	5.96	12	0.39*106	15.4*10–6	6
150 µM	2.43	5.2	5.3	12.79	12.79	14	0.42*106	30.8*10–6	13
150 µM	2.72	4.8	6.95	13.93	13.93	16	0.53*106	27.9*10–6	15
300 µM	4.62	10.75	18.38	23.5	23.5	23	0.37*106	30.0*10–6	11
*Average*							0.42*10^6^	26.1*10^–6^	*11*

Concentrations of the five species in *scheme 1* determined from SEC measurements as described in the text using different initial concentrations (1∶1:1 of Atox1:Cu:WD4) as indicated. Also, the % of the total copper found in hetero-complex is reported. The equilibrium concentrations established are used to derive K_1_ and K_2_ and from this the copper exchange factor K_1_*K_2_ is calculated. Two experiments with 150 µM starting concentrations are reported. For 75 µM, also the opposite reaction, mixing Cu-WD4 with apo-Atox1 was performed.

Using the K_1_ and K_2_ values determined from the SEC experiments we return to **Figure S1** and estimate the amount of all species in the mixture. With this, we can subtract the contributions from the individual proteins and obtain an extinction coefficient for the pure hetero-complex of 9945 M^-1^cm^-1^ at 280 nm. For comparison, extinction coefficient for Cu^2+^ in azurin is 6500 M^–1^cm^–1^ at 530 nm [27] and for a multi-Cu cluster (with 6 to 8 Cu ions) in a metallothionine it is about 110,000 M^–1^cm^–1^ at 270 nm [28]. Using the derived extinction coefficient we analyzed the data in **Figure 3C** and checked if the de-convoluted peak for the hetero-complex corresponds to the amount of protein that was calculated from the Atox1 peak (**Table 1**). A good agreement between the two ways of estimating the hetero-complex concentration was found (±10%), showing that our analysis of SEC is robust.

With defined K_1_ and K_2_, the population of species in the mixture in **Figure 2B** can also be determined. The contributions from the apo- and holo-forms of the two proteins could be subtracted from the measured CD signal to reveal the CD profile for pure hetero-complex. The dotted line in **Figure 2B** is the estimated CD spectrum for pure hetero-complex scaled to the same concentration as in the mixture in **Figure 2B**.

### Enthalpic Contributions from ITC Data

**Figure 4:**
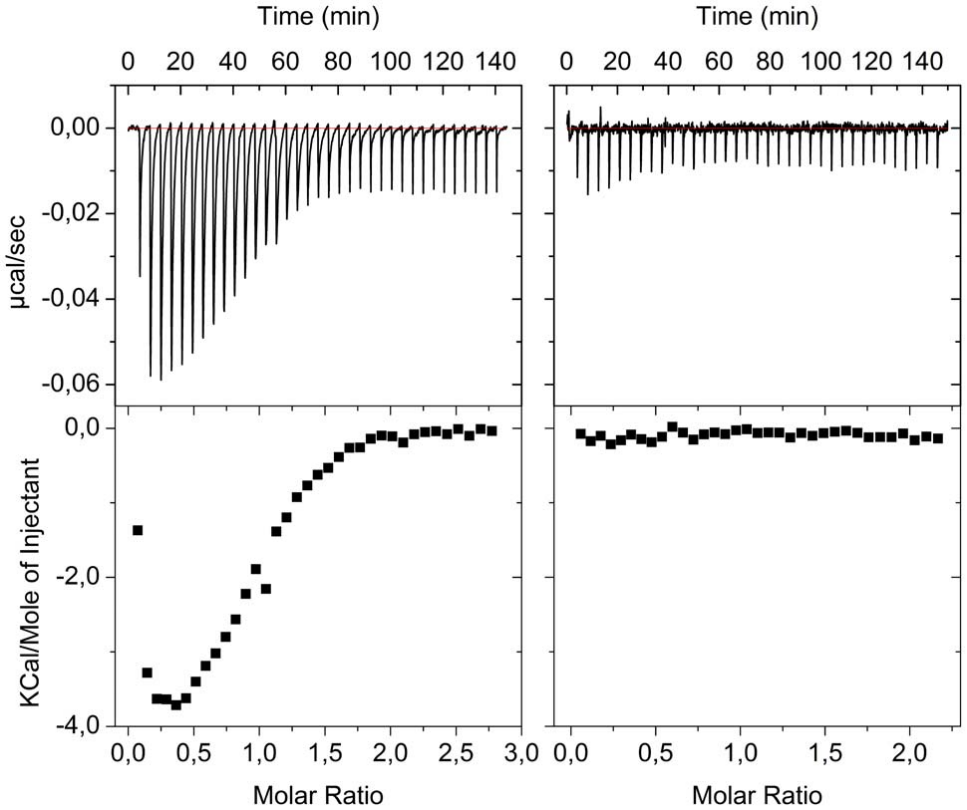
ITC reveals interaction energetics. A. A solution of 666 µM Cu-Atox1 (in the syringe) is titrated into a solution of 52 µM apoWD4 (in the reaction chamber) at 3°C. B. A solution of 666 µM apo-Atox1 (in the syringe) is titrated into a solution of 62 µM apoWD4 (in the reaction chamber) at 3°C. The top plots are the raw data of heats versus time and the bottom plots are integrated heats as a function of molar ratio of Atox1/WD4. Noise estimation based on the data in B predicts uncertainties for individual ITC points of 0.03 kcal/mol.

Isothermal titration calorimetry (ITC) was used to deduce the enthalpic and entropic contributions of the two steps in *scheme 1*. Titration of Cu-Atox1 into a solution of apo-WD results in negative reaction enthalpies (**Figure 4A**). As expected, when apo proteins (**Figure 4B**) are titrated into each other, no reaction heats are observed. To analyze the ITC data we need to relate the heat data to steps 1 and 2 in the equilibrium in *scheme 1*. This can be done considering what happens in each of the injections in **Figure 4A**. For instance, with the first injection of Cu-Atox1 (4 µM) to a fixed amount of apo-WD4 (52 µM), the equilibrium in *scheme 1* is shifted to the right. The first equilibrium is in essence completely shifted towards the hetero-complex as the concentrations are above 1/K_1_ and apo-WD4 is in excess. Thus, the distribution of equilibrium species after this injection will be dictated by K_2_ and the amount of added Cu-Atox1. We can use the K_2_ established in the SEC analysis (**Table 1**) to calculate the extent of step 2 at this condition and it corresponds to 89% (i.e., most of the hetero-complex dissociates as K_2_ is higher than the current concentrations). The integrated enthalpy change per mol (ΔH_a_) detected after the 1^st^ injection thus equals 100%*ΔH_1_+89%*ΔH_2_ (i.e., all molecules goes through step 1 and 89% of these continue to products).

**Figure 5:**
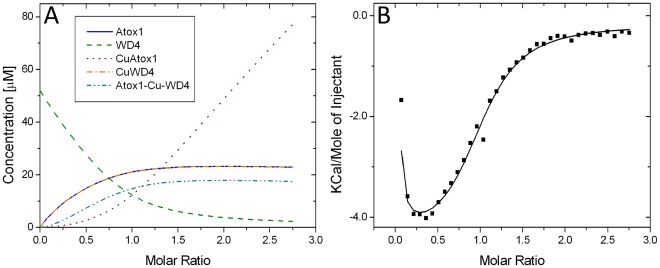
Computer simulations mimic ITC titration. **A.** Concentrations of the five species as a function of progress of the titration experiment shown in Figure 4A using the K values that were derived from the SEC data. **B.** ITC experimental data (from Figure 4A) together with the best fit to the data (see Supplement for details).

Similar reasoning can be made for every point in the titration resulting in an extensive equation system with two unknown parameters (ΔH_1_ and ΔH_2_). Due to the presence of experimental errors in each data point, this problem is best solved numerically via a computer algorithm that fit all points simultaneously. This task was facilitated by a program written in Matlab (see **Data S1**). Using the values of K_1_ and K_2_ derived from SEC we computed the variations of concentrations of the five species in *scheme 1* during the experimental titration in Figure 4A (**Figure 5A**). It emerges that apo-Atox1 and Cu-WD4 builds up more quickly than the hetero-complex. At the end of the titration there are roughly similar amounts of apo-Atox1, Cu-WD4 and hetero-complex and almost no apo-WD4.

We then incorporated the algorithm for computing concentrations (described above) into a program which allows direct fitting of the ITC data in order to derive enthalpy changes and equilibrium constants (**Data S1**). The program accounts for the linked equilibria, the enthalpy change for each step, and the dilution of all concentrations with each injection. In **Figure 5B**, we show the best fit to the ITC data with K_1_, K_2_, ΔH_1_ and ΔH_2_ as floating parameters. In support of an appropriate reaction mechanism, the features of the experimental data are reproduced in the fit.

While the optimal K_1_ and K_2_ from the least-squares procedure did not exactly match the values derived from SEC, the value of K_2_ was identical to that obtained from the SEC analysis and the value of K_1_ differed by less than a factor of four (which can be considered within the experimental uncertainty). The output values of ΔH_1_ and ΔH_2_ from this fit are –25 kJ/mol and+14 kJ/mol, respectively. Thus, formation of the hetero-complex involves favorable (exothermic) enthalpy changes whereas dissociation into products is an endothermic reaction. Combining the ΔG (from SEC data) and ΔH (from ITC data) values, the size of TΔS for each step was calculated (**Table 2**). Hetero-complex formation is accompanied by a small favorable (positive) entropy change whereas disassembly into products involves negative entropy changes. The overall transfer of Cu from Atox1 to WD4 is facilitated by an overall favorable enthalpy change but an overall unfavorable entropy change.

**Table 2:** Thermodynamic parameters for steps 1 and 2, and overall reaction.

	Step 1	Step 2	Overall
ΔH (kJ/mol)	–25.1	+14.0	–11.1
T*ΔS (kJ/mol)	5.0	–10.6	–5.6
ΔG (kJ/mol)	–30.1	+24.6	–5.5
K	0.42*10^6^ M^–1^	26.1*10^–6^ M	11

The parameters are based on *scheme 1* and equilibrium constants from SEC (Table 1) and ITC data, as described in the text.

## Discussion

Many processes in living systems occur through transient, often weak, interactions among proteins. In these cases, the interaction is often associated with a small, negative change in free energy. If the free energy change was large and negative, the complex would be stable and non-transient, whereas if it were positive no interaction would occur. To understand mechanisms of biological activity, studies of the thermodynamics and kinetics of weak, transient protein-protein and protein-ligand interactions are crucial.

Metal homoeostasis in cells is a process involving many transient protein-metal and protein-protein interactions. Cu homoeostasis and trafficking occur through Cu-mediated protein-protein interactions where Cu is bound to ligands from both proteins of the complex. The free energy of formation of Cu-mediated protein-protein complexes is the outcome of the balance of the metal-donor(s) bond energies, of the hydrophobic and hydrophilic interaction energies at the interface, and of the entropic implications of these interactions. Additional factors to consider are solvent effects, both at the metal site and the protein-protein interface, the de-protonation of ligand side chains, and intra-protein structural rearrangements. To avoid protein-protein interactions in absence of metal, the contribution to the change in free energy resulting from protein-protein interactions alone must be positive. Thus, the determining energetic contribution leading to formation of detectable amounts of complex in the presence of metal results from the involvement of amino acid side chains from both proteins in the coordination sphere of Cu. Here, for the first time, the energetic (enthalpic and entropic) contributions for the two elementary steps resulting in Cu transfer from the human chaperone Atox1 to the fourth metal-binding domain of the Wilson disease protein, WD4, have been dissected.

We used SEC as a new approach to derive the equilibrium constants for *scheme 1*. Because WD4 elution is atypical, it was possible to separate the Atox1 peak (which using dual wavelengths for detection can be resolved in apo and holo fractions) from those of WD4 and the hetero-complex. With values on apo- and holo-Atox1 concentrations, the concentrations of all other species could be calculated and thereby K_1_ and K_2_ were defined. We note that in this analysis, it is essential to have concentrations well above the individual K_D_ values for both Cu-Atox1 and Cu-WD4, as we assume that added metal is bound stoichiometrically in a 1∶1 ratio (demonstrated in **Figure S5**). Cu affinities for Atox1 (and homologs in other organisms), MKs and WDs are high; at pH 7 or higher, values of 10^10^ M^–1^ to 10^18^ M^–1^ have been reported [29]. In our SEC experiments, the lowest levels of species present are in the low µM range, thus still 10000-fold above 10^–10^ M.

Our K_1_*K_2_ = K_ex_ value of ∼10 is in good agreement with a published K_ex_ value for Atox1 and WD4 of ∼5 extracted from NMR [20]. Moreover, K_ex_ values for Atox1 and MK2/MK5 are 5–10 [19]. The K_ex_ factor in the yeast system (Atx1 to Ccc2) was reported to be 1.4; i.e., the vectorial gradient is shallower [30]. A K_1_ value of 10^5^, i.e. similar to our result, was reported for Atox1-Cu-MK1 although this value may truly be a combination of K_1_ and 1/K_2_ [16]. K_2_ values (which may truly be combinations with 1/K_1_) of 0.5*10^–6^ for CopZ-Cu-CopA interactions [31] and of 1–19*10^–6^ for Atox1-Cu-MK interactions [32] in Biacore experiments have been reported. Despite minor variations, it appears that making and breaking the Cu-bridged hetero-complex is achieved via affinities in the µM range.

Calorimetry analysis resolved enthalpic contributions of each individual step in *scheme 1*. We found that hetero-complex formation involves negative enthalpy and positive entropy changes. One may speculate that the negative enthalpy comes from favorable (but weak) interactions at the protein-protein surface that forms when Cu interactions bring the two proteins close. Based on data for the Atox1-Ccc2 and Atox1-MK1 complexes, the protein-protein interface in the hetero-complex is relatively small and involves amino acids not normally found at interfaces [16]. A number of favorable electrostatic interactions across the interface have been identified [16], [30]. Increased entropy upon hetero-complex formation may result from structural changes in the target domain. It was reported that when Ccc2 and MK1 formed hetero-complexes with their respective chaperones, helix 1 (which participates in the interface between the proteins) in both Ccc2 and MK1 unwinded [16], [30]. In accord, we found WD4 to be particularly flexible and helix 1 unfolded and refolded during our previous MD simulations of the individual domain [33].

WD4 binds Cu with a ∼5 kJ/mol lower free energy (higher affinity) than Atox1. The increased affinity of WD4 for Cu over Atox1 is due to a more negative enthalpy change of Cu binding in WD4 that is counteracted only in part by a more negative entropy change for Cu binding in WD4. These differences indicate that, apart from the Cu-Cys interactions, there are additional changes upon Cu binding in WD4 that are not found when Cu binds Atox1. In agreement, our earlier computations have demonstrated that since WD4 exhibits more structural dynamics in the apo form as compared to apo-Atox1, Cu binding to WD4 results in more changes in the protein interaction network and reduced structural dynamics as compared to upon Cu binding to Atox1 [33], [34].

In the Atox1-WD4 system, the Cu-bridged hetero-complex is thermodynamically more stable than the isolated proteins (reactants and substrates), which suggest a weakly trapped intermediate. Formation of a long-lived intermediate *in vivo* may be instrumental to determining rearrangements of inter-domain interactions in the ATPase. Such rearrangements of domains during the catalytic cycle appear important for the enzymatic function and for determining the intracellular localization of the ATPase [35], [36]. It is also possible that *in vivo*, ATP hydrolysis may speed up the reaction and change the thermodynamics of the Cu transfer steps from chaperone to target.

## Materials and Methods

### Protein Production

For WD4, residues 356 to 429 in the UniProt entry P35670 (ATP7B_HUMAN) were cloned into a pET24d vector and transformed it into Rosetta 2 cells (Novagen). The cells were grown to OD_600_ of 2, induced with 1 mM IPTG and harvested after 2.5 hours. The cells were lysed using sonication, the lysate run over a HiTrap Q FF column (GE Healthcare) in 25 mM MES buffer at pH 5.7. The WD4 fractions were concentrated and run over a Sephadex S30 column in 40 mM TrisHCl, 50 mM NaCl at pH 7.6. Atox1 was expressed from a pET21 vector transformed into Rosetta 2 cells [37]. The cells were grown to OD_600_ of 2, induced with 1 mM IPTG and harvested after 4 hours. The cells were lysed using sonication, the lysate run over a HiTrap SP FF column (GE Healthcare) in 25 mM MES buffer at pH 5.7. The Atox1 fractions were concentrated and run over a Sephadex S30 in 40 mM TrisHCl, 50 mM NaCl pH 7.6. All purification steps were performed in presence of 2 mM DTT; both proteins elute as apo proteins. Protein purity was confirmed by single bands on SDS-PAGE and mass spectrometry. Concentrations were determined using ε_280_ of 1,500 and 2,980 M^–1^cm^–1^ (based on amino acid sequence) for WD4 and Atox1, respectively. For holo-protein experiments, both Atox1 and WD4 were loaded with stoichiometric amounts of Cu in presence of a 5-fold excess of DTT over Cu to assure reduction of the Cu ion. Stoichiometric 1∶1 binding of Cu to Atox1 and to WD4 was confirmed by near-UV CD and absorption titrations.

### CD

Near-UV CD spectra were collected in a 10 mm quartz cell between 250–380 nm at 20°C (J-810 spectropolarimeter, Jasco). Protein concentration was 50 µM (thus, 50+50 µM in total when the proteins were mixed) in 40 mM TrisHCl, 50 mM NaCl, 500 µM DTT at pH 7.6. Protein mixtures were incubated for 10 minutes at 20°C before analysis (no change in CD was found upon longer incubation times).

### SEC

SEC was performed with a Superdex 75 10/300 analytical column (volume of 24 ml) on an ÄKTA purifier (GE Healthcare) at 4°C. The column was pre-equilibrated with 40 mM TrisHCl, 50 mM NaCl, no DTT at pH 7.6. The protein samples were prepared in the same buffer with the addition of 2mM DTT. Sample was injected with a Hamilton SYN50018P syringe and a 100 µl injection loop. The elution profiles of protein samples were monitored using dual-channel absorption detection at 254 nm and 280 nm. The identity of the proteins found in some elution fractions was assessed by MALDI-MS using a Voyager STR-DE instrument (AB Sciex) and sinapinic acid as matrix (Umeå Protein Analysis Facility, Umeå University). A range of experiments were performed (as described in detail in the text), such as mixture of apo proteins, individual holo-proteins, reaction mixtures at salt concentrations between 50 to 200 mM. Initial protein concentrations ranged from 75 µM to 150 and 300 µM.

### ITC

ITC experiments were performed with an ITC_200_ (MicroCal). In a typical run, 35 automated injections of 1.11 µl with 200–300 s breaks in between injections were made at 3°C and 500 rpm stirring speed in low feedback mode. The cellular protein (apo-WD4) concentrations were varied between 40 and 70 µM while the protein concentration in the syringe (Cu-Atox1; and in control experiments apo-Atox1) was 666 µM for all runs. The buffer was 40 mM TrisHCl, 50 mM NaCl and 2 mM DTT at pH 7.6. Five similar experiments were performed with identical results (within 5%).

### NMR

NMR samples were prepared by dilution of protein stock into a D_2_O-based buffer (40 mM TrisHCl, 50 mM NaCl, 2 mM DTT at pH 7.6). Diffusion coefficients were measured at 6.4^o^C using a DRX600 NMR instrument (Bruker, Inc.) fitted with a triple-resonance 5-mm probe with a z-axis gradient system. A PFG BP-STE sequence from the Bruker library was used with a diffusion delay of 200 ms, bipolar gradient pulses of 4 ms (2×2 ms) and gradient amplitude varying linearly between 7.4 and 33.7 G/cm in 14 steps. All initial data handling was performed in Bruker Topspin 2.0. Linear baseline corrections and integrations of the aliphatic region of interest were performed with scripts in Matlab 6.5 (The MathWorks Inc.). Non-linear least-squares fits with single exponential functions to the integrated protein resonances were executed with the Levenberg-Marquardt algorithm.

### Data Analysis and Simulation

Details in **Data S1**.

## Supporting Information

Figure S1 **Absorption of heterocomplex.** Absorption spectra of individual solutions of 50 µM apo- and holo-forms of WD4 and Atox1, together with the absorption spectrum for a mixture of 50 µM Cu-Atox1 and 50 µM apo-WD4. The absorption is higher at 254 nm for the Cu- versus the apo-forms while the extinction coefficient is unchanged at 280 nm. Using the determined K_1_ and K_2_ values, the amount of each species in the mixture is calculated and from this the contributions to the absorption spectrum of the mixture from the individual proteins are subtracted. The remaining absorption can be used to derive an extinction coefficient for the hetero-complex at 280 of 9945 M^–1^cm^–1^.
(TIF)

Figure S2 **Analysis of heterocomplex. A.** Mass spectrometry was used to analyze the content of SEC elution peaks. The two peaks in the elution profile for a mixture of apo proteins are confirmed to contain only WD4 and Atox1, respectively (black trace, black mass values). When Cu-Atox1 is mixed with apo WD4 (red trace, red mass values) the first peak, which can be decomposed into two underlying peaks, contains both WD4 and Atox1, whereas the second large peak contains only Atox1. (It is not possible to detect Cu forms and hetero-complexes directly via mass spectrometry as these complexes fall apart during the experiment.) For each of Atox1 and WD4 there are two masses corresponding to each protein: one with and one without the first Met residue (131 Da). The error in the detected masses is ±2 Da. **B.** The Atox1-Cu-WD4 hetero-complex was visualized on a native gel. Different combinations of Atox1 (pI 6.7), WD4 (pI 4.0) and Cu were analyzed on a pH 8.8 Tris-Tricine 3–20% native gradient gel. *Lane 1.* Apo-WND4. *Lane 2.* Apo-Atox1. *Lane 3.* 1∶1 mixture of apo-Atox1 and apo-WND4. *Lane 4.* 1∶1:0.5 mixture of apo-Atox1 and apo-WD4 and Cu. *Lane 5.* 1∶1:1 mixture of apo-Atox1 and apo-WD4 and Cu. The position of Atox1, WD4 and the hetero-complex are indicated. As expected, since the complex will have an average pI, the hetero-complex is found in between the positions of the individual proteins. Individual samples of holo-Atox1 and holo-WD4 are found at the same positions as the corresponding apo proteins (data not shown). The smear in lane 5, and the absence of a detectable amount of hetero-complex in lane 4, may be explained by the different forces acting on the two proteins in the hetero-complex due to their different pIs causing some complex dissociation when running on the gel.
(TIF)

Figure S3 **SEC analysis as a function of concentration.** SEC of mixtures of 75 (top), 150 (center) and 300 (bottom) µM Atox1 and equal amounts of WD4 in apo- or Cu-loaded forms as indicated. For each starting concentration, one experiment with only apo proteins and one experiment with a mixture Cu-Atox1 and apo-WD4 were analyzed. For the lowest protein concentration, an additional experiment with apo-Atox1 and Cu-WD4 (i.e. opposite reaction) was also investigated (light blue, top panel).
(TIF)

Figure S4 **CD spectra of heterocpomplex.** Near-UV CD spectra of 1∶1 mixtures of Cu-Atox1 (50 µM) and WD4 (50 µM) at three different NaCl concentrations (50, 100 and 400 mM). In agreement with the reported SEC data, the amount of hetero-complex (i.e., Atox1-Cu-WD4) detected (based on its CD characteristics) is independent of the salt concentration. For comparison the theoretical CD signal derived for no reaction (i.e., sum of Cu-Atox1 and apo-WD4 signals) and for 100% reaction (i.e., sum of signals for apo-Atox1 and holo-WD4) are also shown.
(TIF)

Figure S5 **Probing protein:Cu stoichiometries using absorption.** A number of mixtures of a fixed amount of Atox1 (or WD4) and various amounts of Cu between 0 and 1.5 times the protein concentration were purified from unbound Cu individually via SEC. The absorption of the resulting protein peak was analyzed at 254 and 280 nm. Whereas the protein absorbs at 280 nm, bound Cu absorbs at 254 nm (see Figure S1). Below, the 254/280 nm absorption ratio is plotted as a function of mixing ratio of Cu-to-protein (A. Atox1; B. WD4). For both proteins, the absorption increases in essence linearly until a stoichiometry of 1∶1 is reached. This confirms stoichiomteric 1∶1 binding with a high affinity. The use of SEC prior to analysis enables elimination of DTT-Cu complexes that may be present in samples where the protein is saturated with metal; these complexes also absorb at 254 nm, which complicates the analysis.
(TIF)

Table S1 **Probing WD4 by NMR.** Diffusion coefficients estimated from NMR measurements for apo- and holo-WD4 at low and high salt concentrations. The expected diffusion coefficient, calculated from first principles using Hydropro7.C (Biophys. J. 78, 719–730 (2000)) using the crystal structure of Atox1 is 7.4*10^–11^ m^2^/s at the temperature of measurement (6.4°C). Reported uncertainties are standard deviations from non-linear fits to the data.
(DOCX)

Data S1 **Data analysis and simulation details.**
(DOCX)
