# Supplementary figures and images for: In Vitro Thermodynamic Dissection of Human Copper Transfer from Chaperone to Target Protein
Source: PLoS One. 2012 May 4;7(5):e36102. doi: 10.1371/journal.pone.0036102 (PMC3344837; doi:10.1371/journal.pone.0036102)

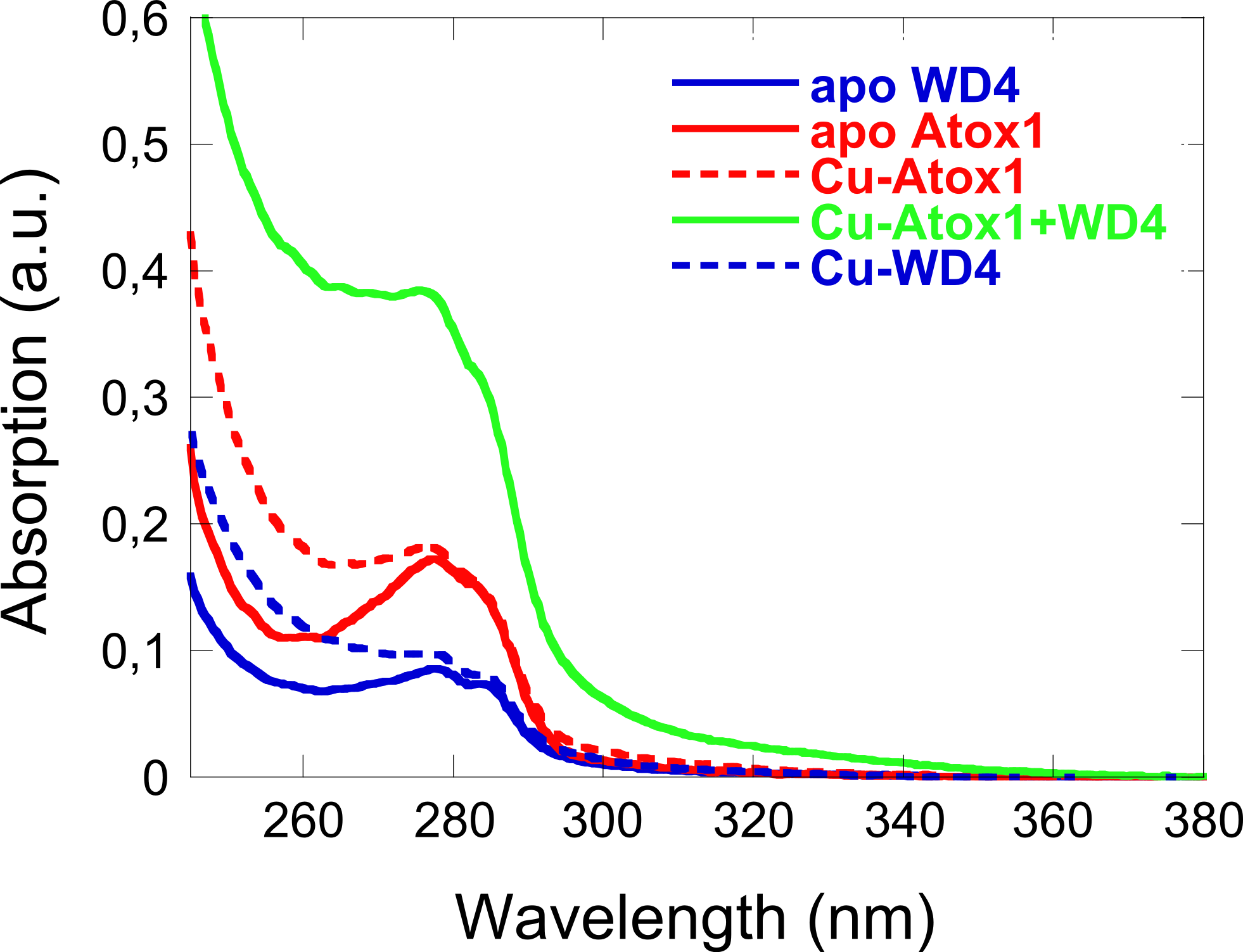

Supplement: Figure S1 — Absorption of heterocomplex. Absorption spectra of individual solutions of 50 µM apo- and holo-forms of WD4 and Atox1, together with the absorption spectrum for a mixture of 50 µM Cu-Atox1 and 50 µM apo-WD4. The absorption is higher at 254 nm for the Cu- versus the apo-forms while the extinction coefficient is unchanged at 280 nm. Using the determined K1 and K2 values, the amount of each species in the mixture is calculated and from this the contributions to the absorption spectrum of the mixture from the individual proteins are subtracted. The remaining absorption can be used to derive an extinction coefficient for the hetero-complex at 280 of 9945 M–1cm–1. (TIF) [file pone.0036102.s001.tif]

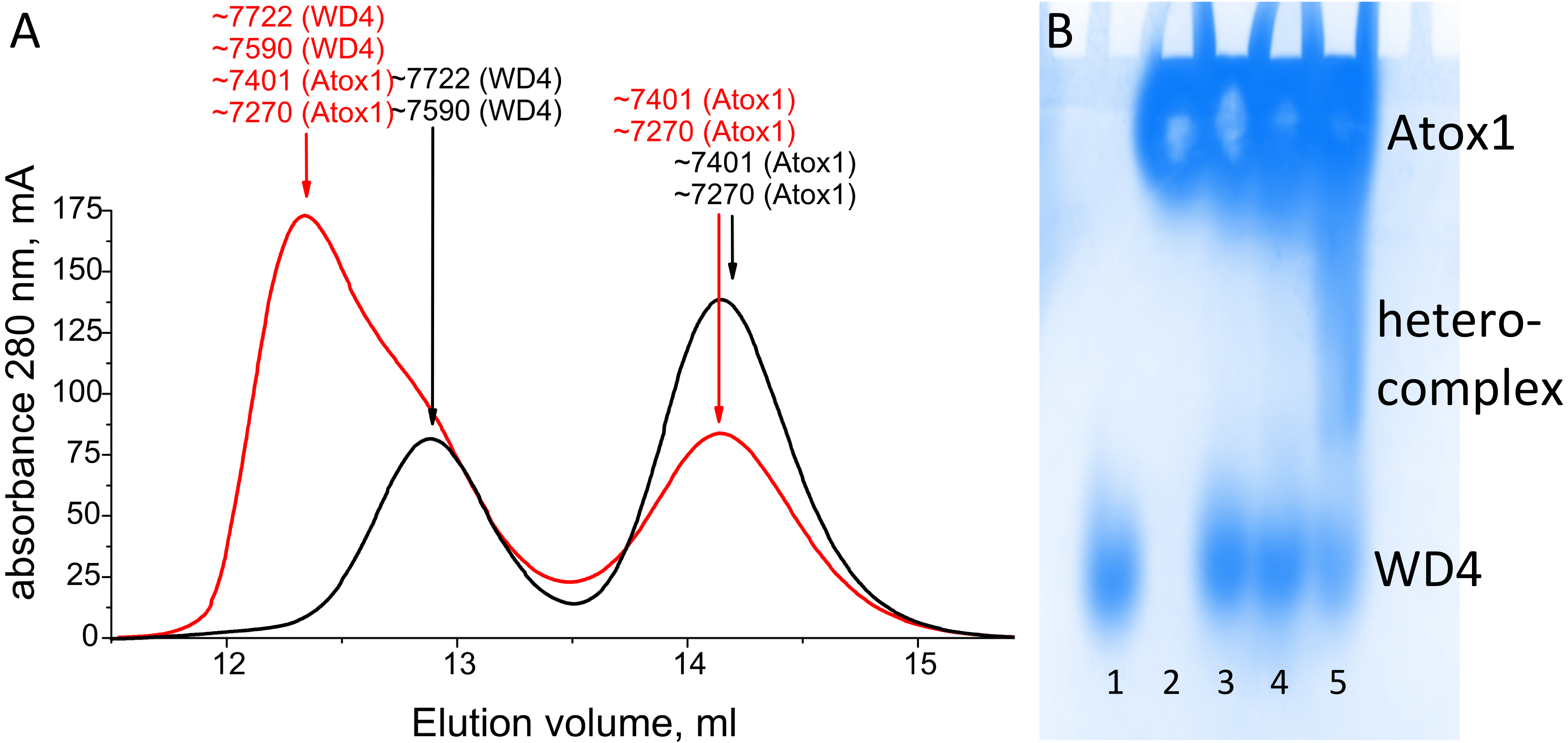

Supplement: Figure S2 — Analysis of heterocomplex. A. Mass spectrometry was used to analyze the content of SEC elution peaks. The two peaks in the elution profile for a mixture of apo proteins are confirmed to contain only WD4 and Atox1, respectively (black trace, black mass values). When Cu-Atox1 is mixed with apo WD4 (red trace, red mass values) the first peak, which can be decomposed into two underlying peaks, contains both WD4 and Atox1, whereas the second large peak contains only Atox1. (It is not possible to detect Cu forms and hetero-complexes directly via mass spectrometry as these complexes fall apart during the experiment.) For each of Atox1 and WD4 there are two masses corresponding to each protein: one with and one without the first Met residue (131 Da). The error in the detected masses is ±2 Da. B. The Atox1-Cu-WD4 hetero-complex was visualized on a native gel. Different combinations of Atox1 (pI 6.7), WD4 (pI 4.0) and Cu were analyzed on a pH 8.8 Tris-Tricine 3–20% native gradient gel. Lane 1. Apo-WND4. Lane 2. Apo-Atox1. Lane 3. 1∶1 mixture of apo-Atox1 and apo-WND4. Lane 4. 1∶1:0.5 mixture of apo-Atox1 and apo-WD4 and Cu. Lane 5. 1∶1:1 mixture of apo-Atox1 and apo-WD4 and Cu. The position of Atox1, WD4 and the hetero-complex are indicated. As expected, since the complex will have an average pI, the hetero-complex is found in between the positions of the individual proteins. Individual samples of holo-Atox1 and holo-WD4 are found at the same positions as the corresponding apo proteins (data not shown). The smear in lane 5, and the absence of a detectable amount of hetero-complex in lane 4, may be explained by the different forces acting on the two proteins in the hetero-complex due to their different pIs causing some complex dissociation when running on the gel. (TIF) [file pone.0036102.s002.tif]

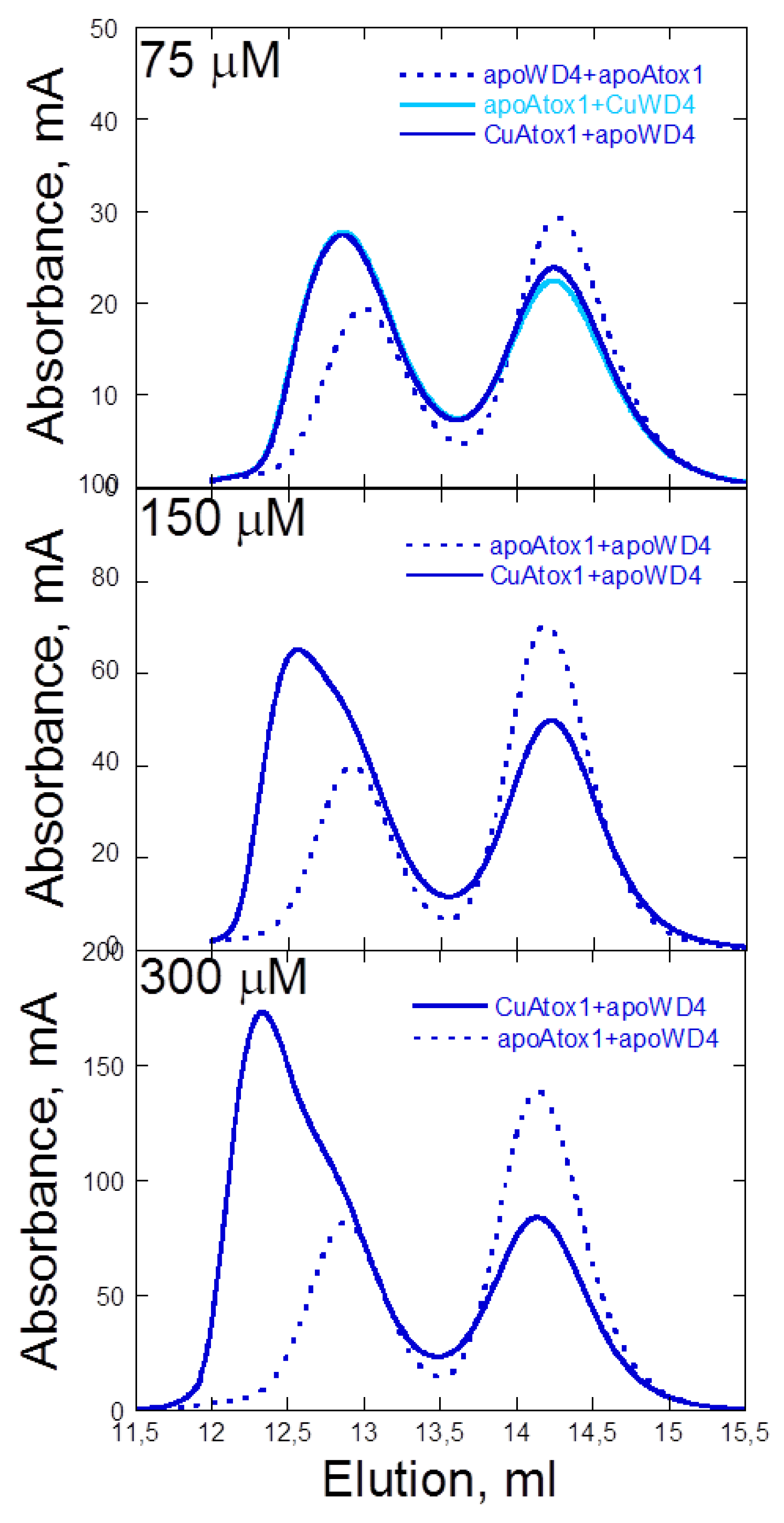

Supplement: Figure S3 — SEC analysis as a function of concentration. SEC of mixtures of 75 (top), 150 (center) and 300 (bottom) µM Atox1 and equal amounts of WD4 in apo- or Cu-loaded forms as indicated. For each starting concentration, one experiment with only apo proteins and one experiment with a mixture Cu-Atox1 and apo-WD4 were analyzed. For the lowest protein concentration, an additional experiment with apo-Atox1 and Cu-WD4 (i.e. opposite reaction) was also investigated (light blue, top panel). (TIF) [file pone.0036102.s003.tif]

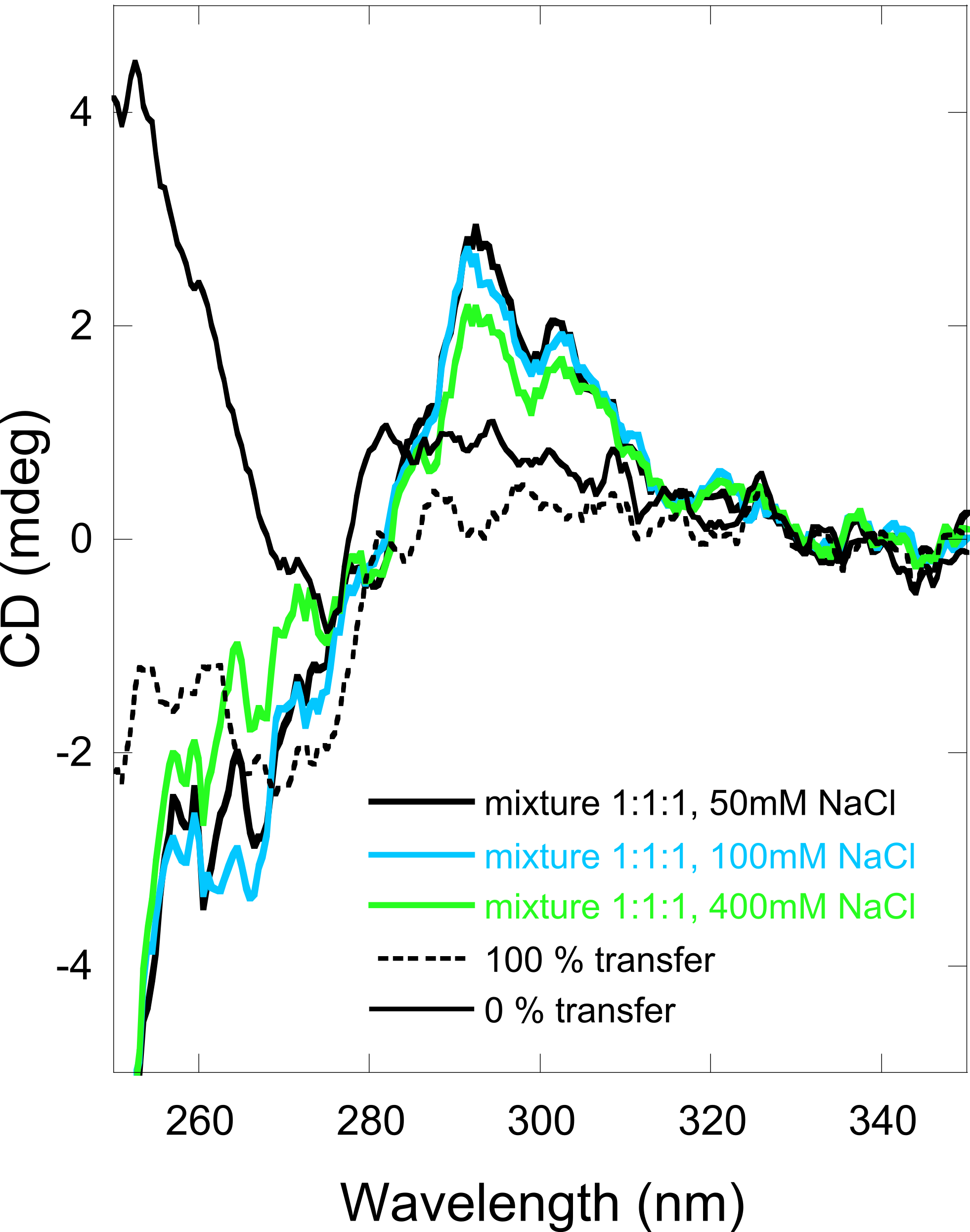

Supplement: Figure S4 — CD spectra of heterocpomplex. Near-UV CD spectra of 1∶1 mixtures of Cu-Atox1 (50 µM) and WD4 (50 µM) at three different NaCl concentrations (50, 100 and 400 mM). In agreement with the reported SEC data, the amount of hetero-complex (i.e., Atox1-Cu-WD4) detected (based on its CD characteristics) is independent of the salt concentration. For comparison the theoretical CD signal derived for no reaction (i.e., sum of Cu-Atox1 and apo-WD4 signals) and for 100% reaction (i.e., sum of signals for apo-Atox1 and holo-WD4) are also shown. (TIF) [file pone.0036102.s004.tif]

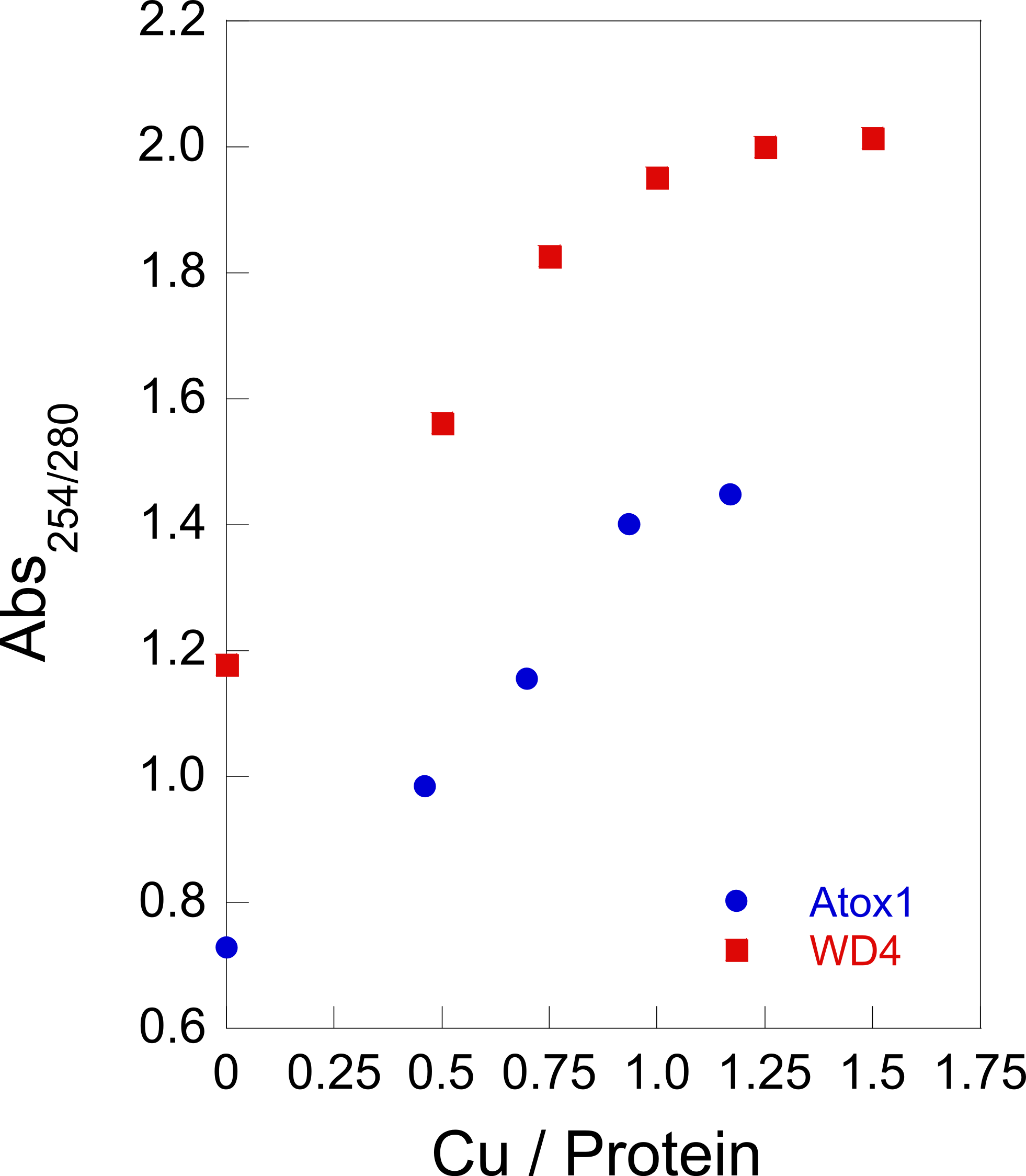

Supplement: Figure S5 — Probing protein:Cu stoichiometries using absorption. A number of mixtures of a fixed amount of Atox1 (or WD4) and various amounts of Cu between 0 and 1.5 times the protein concentration were purified from unbound Cu individually via SEC. The absorption of the resulting protein peak was analyzed at 254 and 280 nm. Whereas the protein absorbs at 280 nm, bound Cu absorbs at 254 nm (see Figure S1). Below, the 254/280 nm absorption ratio is plotted as a function of mixing ratio of Cu-to-protein (A. Atox1; B. WD4). For both proteins, the absorption increases in essence linearly until a stoichiometry of 1∶1 is reached. This confirms stoichiomteric 1∶1 binding with a high affinity. The use of SEC prior to analysis enables elimination of DTT-Cu complexes that may be present in samples where the protein is saturated with metal; these complexes also absorb at 254 nm, which complicates the analysis. (TIF) [file pone.0036102.s005.tif]
