# Supplementary material for: In Vitro Thermodynamic Dissection of Human Copper Transfer from Chaperone to Target Protein
Source: PLoS One. 2012 May 4;7(5):e36102. doi: 10.1371/journal.pone.0036102 (PMC3344837; doi:10.1371/journal.pone.0036102)
Supplement: Table S1 — Probing WD4 by NMR. Diffusion coefficients estimated from NMR measurements for apo- and holo-WD4 at low and high salt concentrations. The expected diffusion coefficient, calculated from first principles using Hydropro7.C (Biophys. J. 78, 719–730 (2000)) using the crystal structure of Atox1 is 7.4*10–11 m2/s at the temperature of measurement (6.4°C). Reported uncertainties are standard deviations from non-linear fits to the data. (DOCX) [file pone.0036102.s006.docx]

**Table S1.**

|  | **Low salt (*10^-11^ m^2^/s)** | **High salt (*10^-11^ m^2^/s)** |
| --- | --- | --- |
| **Apo-WD4** | **7.53±0.06** | **7.72±0.10** |
| **Holo-WD4** | **7.58±0.05** | **7.67±0.08** |
