# Supplementary material for: In Vitro Thermodynamic Dissection of Human Copper Transfer from Chaperone to Target Protein
Source: PLoS One. 2012 May 4;7(5):e36102. doi: 10.1371/journal.pone.0036102 (PMC3344837; doi:10.1371/journal.pone.0036102)
Supplement: Data S1 — Data analysis and simulation details. (DOCX) [file pone.0036102.s007.docx]

**Data S1. Data analysis and simulation details.**

Experimental ITC thermograms were analyzed computationally assuming transfer of Cu from Atox1 to WD4 via a hetero-complex intermediate (*Scheme 1* in the regular text),

$$Cu-Atox1+WD4 \begin{matrix} \rightleftharpoons\\ K_{1}{\Delta H}_{1} \end{matrix} Atox1-Cu-WD4 \begin{matrix} \rightleftharpoons\\ K_{2}{\Delta H}_{2} \end{matrix} Atox1+Cu-WD4$$

K_1_ and K_2_ are equilibrium constants, and ΔH_1_ and ΔH_2_ are the enthalpy changes, for the individual reaction steps. Dilution effects upon addition of titrant were accounted for during computation of the concentrations of all five species. The total concentration of protein titrated into the ITC sample chamber (*i.e.,* Atox1 in all possible forms; apo, holo, and heterocomplex) was computed as:

$$L_{i}=\left\{ L_{i-1}\left( V_{0}-V_{t} \right)+L_{t}V_{t} \right\}/{V_{0}}$$

where L_i-1_ and L_i_ are the concentrations before and after the current titration step, L_t_ is the concentration of titrant, and V_t_ and V_0_ are the volumes of titrant and of the chamber, respectively. This dilution model effectively assumes that the mixing is slow, *i.e.* the ejected volume (to keep the chamber volume constant during the titration) has concentration L_i-1_ and is thus not considered to mix with the added volume before ejection. The total concentration P_i_ of the protein in the chamber (WD4 in all possible forms; apo, holo and heterocomplex) was similarly calculated as:

$$P_{i}={P_{i-1}\left( V_{0}-V_{t} \right)}/{V_{0}}$$

Using the total concentrations of Cu and the two proteins at each condition and given values for the two equilibrium constants, the equilibrium concentration of each of the five species after each injection can be calculated by solving the mass balance equations. This approach resulted in five coupled non-linear equations that were combined and rearranged algebraically yielding one 4^th^ degree polynomial that was solved numerically using Matlab.

The heat released upon each injection during the ITC titration was computed as:

$$q_{i}=\Delta H_{1}\left( \left[ WD4 \right]_{i-1}-\left[ WD4 \right]_{i} \right)+ \Delta H_{2}\left( \left[ Atox1 \right]_{i}-\left[ Atox1 \right]_{i-1} \right)$$

where the first term describes step 1 of the reaction resulting in WD4 depletion, and the second term reports on the progress of step 2 following the injection. A correction for background heat of dilution was included via a constant offset parameter Q_off_. The first injection in the experimental data sets was excluded from the fits. The experimental ITC curves were fitted using a non-linear least-squares algorithm (Levenberg-Marquardt minimization) in Matlab with K_1_, K_2_, ΔH_1_, ΔH_2_ and Q_off_ parameters floating.
